# Digital Voice Journaling for Depressive Symptoms in Youth: Predicting Symptom Changes Using Machine Learning

**DOI:** 10.3390/healthcare14193238

**Published:** 2026-10-01

**Authors:** Dorothy Ka Ki Ku, Alyssa Yuet Chin Wong, Kit Ying Chan, Yun Kwok Wing, Tim Man Ho Li

**Affiliations:** 1 Li Chiu Kong Family Sleep Assessment Unit, Department of Psychiatry, The Chinese University of Hong Kong, Hong Kong SAR, China; 2 Li Ka Shing Institute of Health Sciences, Faculty of Medicine, The Chinese University of Hong Kong, Hong Kong SAR, China; 3 Gerald Choa Neuroscience Institute, The Chinese University of Hong Kong, Hong Kong SAR, China

**Keywords:** machine learning, journaling, depression, youth

## Abstract

Background/Objectives: Given the existing evidence supporting the benefits of journaling, digital voice journaling may represent a promising approach for alleviating depressive symptoms. This study examined changes in depressive symptoms associated with its use, identified linguistic and acoustic correlates of symptom improvement, and developed machine learning models to predict subsequent improvement. Methods: The study employed a pretest–posttest design. Young adults aged 18–24 years with depressive symptoms (17-item Hamilton Depression Rating Scale [HAMD-17] ≥ 8) completed a seven-day digital voice journaling with twice-daily prompts, followed by a repeated HAMD-17 assessment. Linguistic and acoustic features extracted from the recordings were used to develop 12 machine learning models through five-fold cross-validation, for predicting symptom remission (post-journaling HAMD-17 < 8) and improvement (reduction in severity category), and model performance is evaluated using pooled AUC. Results: Among the 68 participants (age = 20.78 ± 1.98 years; 85.29% female), a mean decrease of 4.54 points in HAMD-17 scores was found with a moderate effect size (Cohen’s d = 0.78). Improvement and remission were observed in 61.76% (*n* = 42) and 52.94% (*n* = 36) of participants, respectively. Multiple linguistic and acoustic features, including achievement-related words, function words, F2 formant frequency, and MFCC 2, were significantly associated with these outcomes. The highest-performing models achieved AUCs of 0.86 (95% CI: 0.76–0.94) for improvement and 0.88 (95% CI: 0.79–0.95) for remission. Conclusions: Depressive symptoms decreased during the seven-day voice journaling period, although the uncontrolled design limits causal interpretation of this change. Furthermore, linguistic and acoustic features may help identify individuals who are more likely to experience symptom improvement or remission.

## 1. Introduction

Depression is a major public health concern, especially in young people. Global prevalences of 33.6% in college students [1] and 27.4% in adolescents aged 10–18 [2] have been reported in recent systematic reviews. In Hong Kong, the prevalence of depressive symptoms has been estimated to be 68.5% and 62.3% in university and secondary school students, respectively [3,4]. These high prevalence rates highlight the need for accessible and scalable strategies to identify and reduce depressive symptoms in youth.

Journaling has emerged as a promising self-help approach for depression, particularly given that many young people routinely express their thoughts and emotions through diaries and social media. It has been proposed that journaling facilitates cognitive processing of distress and integrates emotional experiences into coherent narratives [5,6]. For instance, expressive writing, a journaling practice involving 10–30 min of daily writing about one’s deepest thoughts and feelings over several days, has demonstrated benefits in alleviating depressive symptoms [7,8,9,10]. A recent meta-analysis has reported small-to-moderate improvements in depressive symptoms compared to control conditions [11].

Advances in digital technology have expanded journaling beyond traditional written formats to include voice journaling. Compared with writing, spoken expression is often more spontaneous and less cognitively demanding, potentially facilitating greater emotional disclosure [12,13]. Importantly, voice journaling captures a range of linguistic and acoustic features (e.g., pitch, loudness, and speech rate) that have been associated with depressive symptoms [14,15,16,17,18,19,20]. Recent studies have begun to explore voice journaling for depression assessment, demonstrating that linguistic and acoustic features extracted from voice journaling can detect depressive symptom severity with promising accuracy [21,22]. However, despite growing interest in its assessment potential, little research has examined whether engagement in voice journaling is associated with improvements in depressive symptoms.

Identifying markers of symptom improvement may facilitate progress monitoring and inform personalized care. Recent advances in natural language processing (NLP) and machine learning have enabled the prediction of depression using linguistic and acoustic features derived from speech and text [23,24,25,26,27]. However, most existing research has focused on cross-sectional detection rather than longitudinal tracking of symptom trajectories. Moreover, few studies have examined whether multimodal linguistic and acoustic features captured through voice journaling can predict subsequent improvement in depressive symptoms or remission.

Although linguistic and acoustic features have been linked to depression, the potential utility of digital voice journaling and the predictive value of multimodal features derived from journal entries remain unclear. This study examined changes in depressive symptoms over a seven-day period of digital voice journaling and explored associations between linguistic and acoustic features extracted from journal recordings and subsequent symptom outcomes. We hypothesized that (1) depressive symptom severity would decrease between pre- and post-journaling assessments, (2) exploratory analyses would find linguistic and acoustic features associated with symptom improvement and remission, and (3) machine learning models integrating these features would predict symptom outcomes.

## 2. Materials and Methods

### 2.1. Study Design

This pilot pretest–posttest study recruited participants within the community using convenient sampling through institutional mass emails and social media advertisements from September 2024 to June 2025. The inclusion criterion was native Cantonese speakers aged 18–24 years old and the presence of depressive symptoms, as measured by the 17-item Hamilton Depression Rating Scale (HAMD-17) interview with score of 8 or above [28]. Participants were excluded if they had any psychiatric diagnoses apart from depressive and anxiety disorders, or current or historic voice, speech and language disorders. Psychiatric diagnoses were assessed using the Structured Clinical Interview for DSM-5 Disorders–clinician version (SCID-5-CV) [29]. Participants completed a seven-day digital voice journaling and underwent a post-journaling HAMD-17 assessment. All interviews were conducted via videoconferencing. Ethical approval was obtained from the Joint Chinese University of Hong Kong–New Territories East Cluster clinical research ethics committee (reference no.: 2023.536), and informed consent was obtained from all subjects involved in the study. The study was reported in accordance with the Transparent Reporting of a Multivariable Prediction Model for Individual Prognosis or Diagnosis using Artificial Intelligence (TRIPOD+AI) guidance (Supplementary Data S1 and S2) [30].

### 2.2. Outcome Measures

The presence and severity of depressive symptoms were assessed in two interviews by trained researchers, the day before the voice journaling period, and within seven days after, with the interviewer blinded to the initial assessment and journaling results. HAMD-17 was used, which includes nine items scored from 0 to 4 and eight items scored from 0 to 2, yielding a total score range of 0–52. A score of 8 or above indicates the presence of depressive symptoms and are classified as mild (8–16), moderate (17–23), or severe (≥24) [31]. Chinese-translated versions of the instruments were administered during the interviews [32]. Two binary outcomes were derived from the HAMD-17 scores: (1) remission status (remitted vs. non-remitted), with remission defined as a post-journaling HAMD-17 score below 8; and (2) improvement status (improved vs. non-improved), with improvement defined as a reduction in depression severity category.

### 2.3. Digital Voice Journaling

Participants completed a seven-day digital voice journaling using a Telegram bot. The system automatically delivered guided prompts twice daily, once in the morning and once in the evening. At each session, participants were asked to provide voice-recorded responses regarding (a) their current mood, (b) feelings of guilt related to things they had done or left undone, (c) experiences of anxiety, tension, or irritability, and (d) any physical symptoms, such as dry mouth, indigestion, palpitations, headaches, or sweating, together with possible explanations for these symptoms. These prompts were selected because they target emotional and somatic experiences commonly associated with depression. Speech samples were extracted from each recorded response for analysis.

### 2.4. Feature Extraction

Recordings collected in .mp3 format were standardized by removing silence at the start and end of the audio and using Demucs to remove background noise, defined by any audio softer than −45 dBFS lasting for at least 250 milliseconds, isolating the human vocal track. Prior to speech feature extraction, recordings from the same session were merged, and were only excluded from analysis if the merged audio could not be transcribed. Eighty acoustic features were recorded by OpenSMILE (version 2.5.1), a software which automatically extracts acoustic features and statistical functionals from each voice recording. Some examples of acoustic features include the fundamental frequency of the voice (F0), which is closely related to perceived pitch; the first three formant frequencies (F1–F3), which reflect resonances of the vocal tract and contribute to the acoustic characteristics of speech sounds [33]; and the Mel-Frequency Cepstral Coefficients (MFCC), which capture the spectral properties of speech signals [34]. All voice recordings were transcribed automatically by Tencent Cloud ASR, and words were categorized into 84 psychologically and linguistically meaningful categories, such as emotion words, pronouns, and social words, using the Chinese Linguistic Inquiry and Word Count Dictionary 2015 (CLIWC) [35]. For each acoustic and linguistic feature, the mean, standard deviation (SD), and root mean square of successive differences (RMSSD) were calculated across all recordings to capture overall levels, variability, and short-term fluctuations, respectively. Missing data were handled using complete-case analysis.

### 2.5. Machine Learning Modeling

Twelve machine learning algorithms, including Decision Tree, Random Forest, Support Vector Machine, K-Nearest Neighbors, Logistic Regression, Stochastic Gradient Descent, Multilayer Perceptron, Gaussian Process, AdaBoost, Gradient Boosting, Naïve Bayes, and Quadratic Discriminant Analysis, were implemented in Python (v3.11). Input features included acoustic and linguistic features, demographics, and initial HAMD-17 scores. Model performance was evaluated using five-fold cross-validation. Within each training set, feature selection was conducted using SelectKBest with ANOVA F-statistics (f_classif), retaining the 100 most statistically significant features. To address class imbalance, the Synthetic Minority Over-Sampling Technique (SMOTE) was applied to the training data. Hyperparameters were optimized using GridSearchCV with nested five-fold cross-validation. Features were standardized using StandardScaler, and to prevent data leakage, scaling parameters were computed strictly on the training dataset and applied to the test dataset without refitting. Model performance was assessed using the area under the receiver operating characteristic curve (AUC), accuracy, F1 score, sensitivity, specificity, positive predictive value (PPV), and negative predictive value (NPV). Predicted probabilities from the outer test folds were pooled to calculate a cross-validated AUC. Ninety-five percent confidence intervals for the AUC were estimated using 2000 bootstrap resamples of the pooled out-of-sample predictions. The lower and upper bounds corresponded to the 2.5th and 97.5th percentiles of the bootstrap distribution. Statistical significance was assessed by comparing the bootstrap of AUC distribution against the null value of 0.5 (chance discrimination), with 1.0 indicating perfect discrimination. The performance of multimodal models was also compared with that of demographic-, linguistic-, and acoustic-based models to determine whether integrating multiple data modalities improved prediction accuracy.

### 2.6. Statistical Analysis

Statistical analyses were conducted in R (version 4.4.1), with statistical significance set at *p* < 0.05. Continuous variables are reported as mean ± SD and categorical variables as n (%). Group differences were assessed using independent-samples *t*-tests or Mann–Whitney U tests for continuous variables and chi-square or Fisher’s exact tests for categorical variables. Normality was evaluated using the Shapiro–Wilk test. Changes in HAMD-17 scores from pre- to post-assessment were examined using paired-samples *t*-tests, with Cohen’s d calculated as the effect size.

To examine the associations between individual speech-derived features and the binary outcomes, exploratory binary Logistic Regression analyses were performed. A generalized estimating equation (GEE) model was used to examine whether longitudinal changes in linguistic and acoustic features differed between remitted and non-remitted participants, as well as between improved and non-improved participants, over the 7-day journaling period. The model included fixed effects for day, remission status, and their interaction, adjusting for age, sex, and baseline depression severity (HAMD-17). To account for within-subject correlation across repeated daily observations, an autoregressive (AR [1]) working correlation structure was specified.

## 3. Results

### 3.1. Participant Characteristics

**Figure 1:**
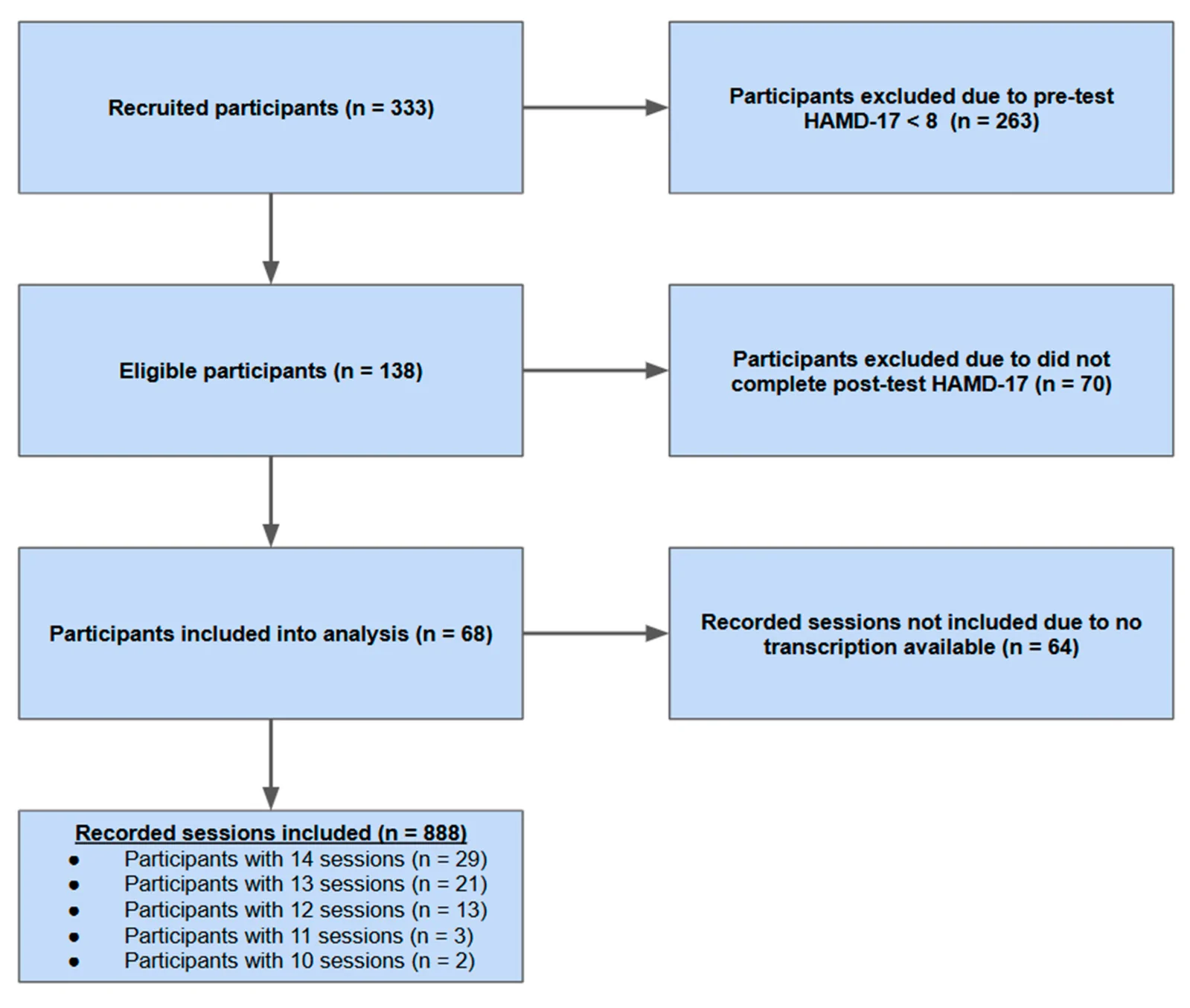
Flowchart of how the number of participants and recordings is reached.

As shown in Figure 1, 68 participants, with an average of 13.06 recorded sessions per participant, were included in this study, of which 58 (85.29%) were female and the mean age was 20.78 ± 1.98 years (Table 1). Over two-thirds of the participants met the criteria for at least one type of depressive or anxiety disorder. Apart from a significantly lower prevalence of anxiety disorders present in the remitted group compared to the non-remitted group, there were no other significant demographic results noted between groups.

### 3.2. Changes in Depressive Symptoms

**Figure 2:**
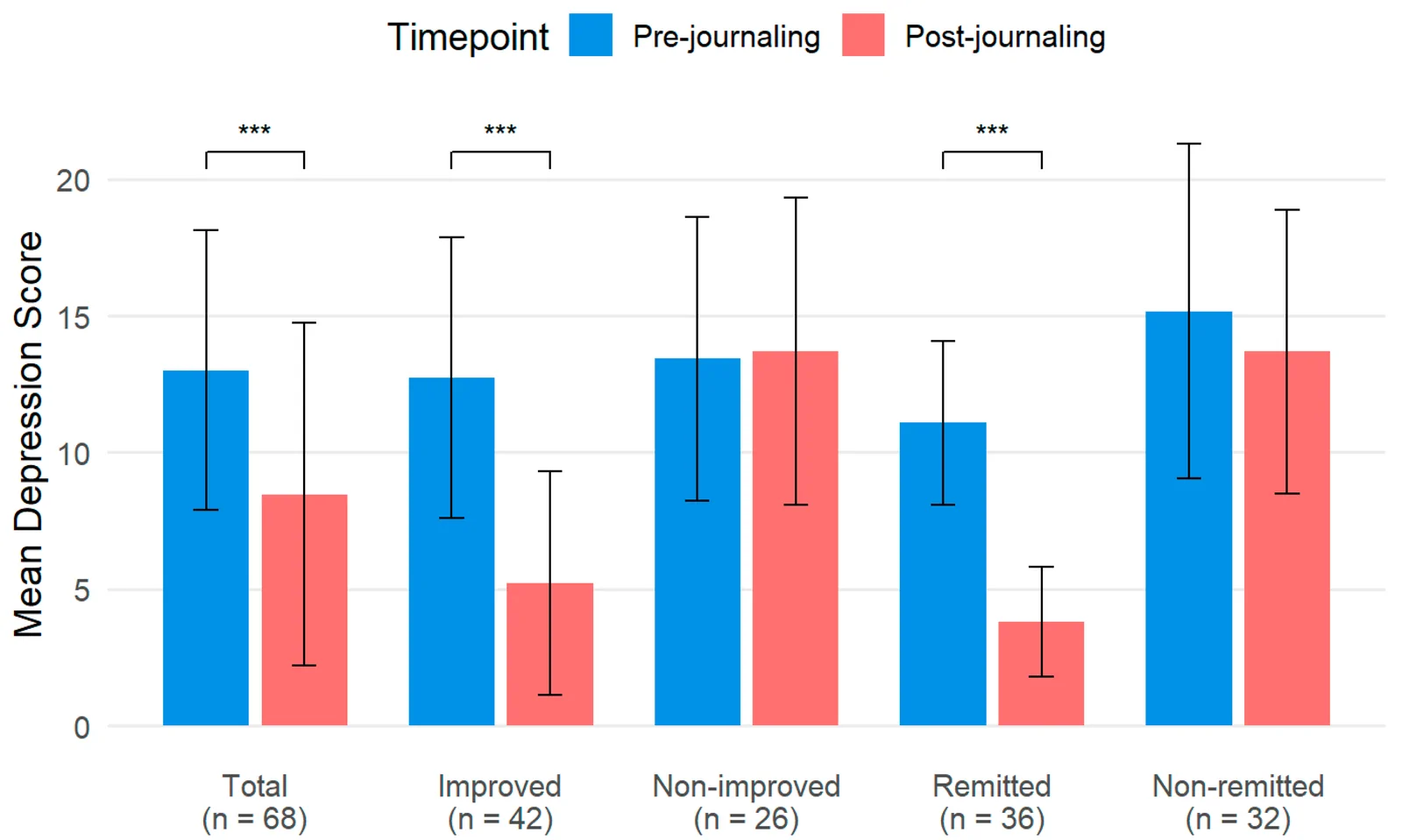
Mean HAMD-17 scores pre- and post-journaling (*** *p* < 0.001).

The Shapiro–Wilk test indicated that the data were normally distributed (W = 1.00, *p* = 0.20). As shown in Figure 2, post-journaling HAMD-17 scores (8.46 ± 6.27) were significantly lower than pre-journaling scores (13.00 ± 5.12). This mean reduction of 4.54 points (95% CI [3.22, 5.86]) was statistically significant, t(67) = 7.00, *p* < 0.001, with Cohen’s d effect size analysis showing a moderate effect size (d = 0.78).

Forty-two (61.76%) participants showed a reduction in depressive scores post-journaling, in which 36 (52.94%) fell under the HAMD-17 depression cut-off score of 8 (Table 2). As shown in Table 2, when further broken down into depression severity categories, 55 (80.88%) of our participants started with a mild level, nine (13.24%) with moderate and four (5.88%) with severe. In those with a pre-journaling level of mild depression severity, 34 (61.82%) showed improvement and remission; in the moderate group, a total of five (55.56%) improved, of which two (22.22%) were remitted; and for the severe group, a total of three (75.00%) had improved at least one level, with no participants achieving remission.

### 3.3. Associations of Speech Features with Depression Outcomes in Exploratory Binary Regression Analyses

Six linguistic and four acoustic features were significantly associated with remission of depressive symptoms (Table 3). Specifically, increased use of achievement-related words, function words, impersonal pronouns, past-tense markers, and home- and work-related terms was associated with a lower likelihood of remission. Conversely, higher F2 and F3 formant frequencies and MFCC 2 values were associated with a greater likelihood of remission, whereas higher loudness was associated with a reduced likelihood of remission.

In addition, four linguistic and two acoustic features were significantly associated with symptom improvement (Table 4). Greater use of achievement-related words, auxiliary verbs, function words, and sadness-related words was associated with a lower likelihood of improvement. In contrast, higher F2 formant frequency and MFCC 2 values were associated with a greater likelihood of improvement.

**Figure 3:**
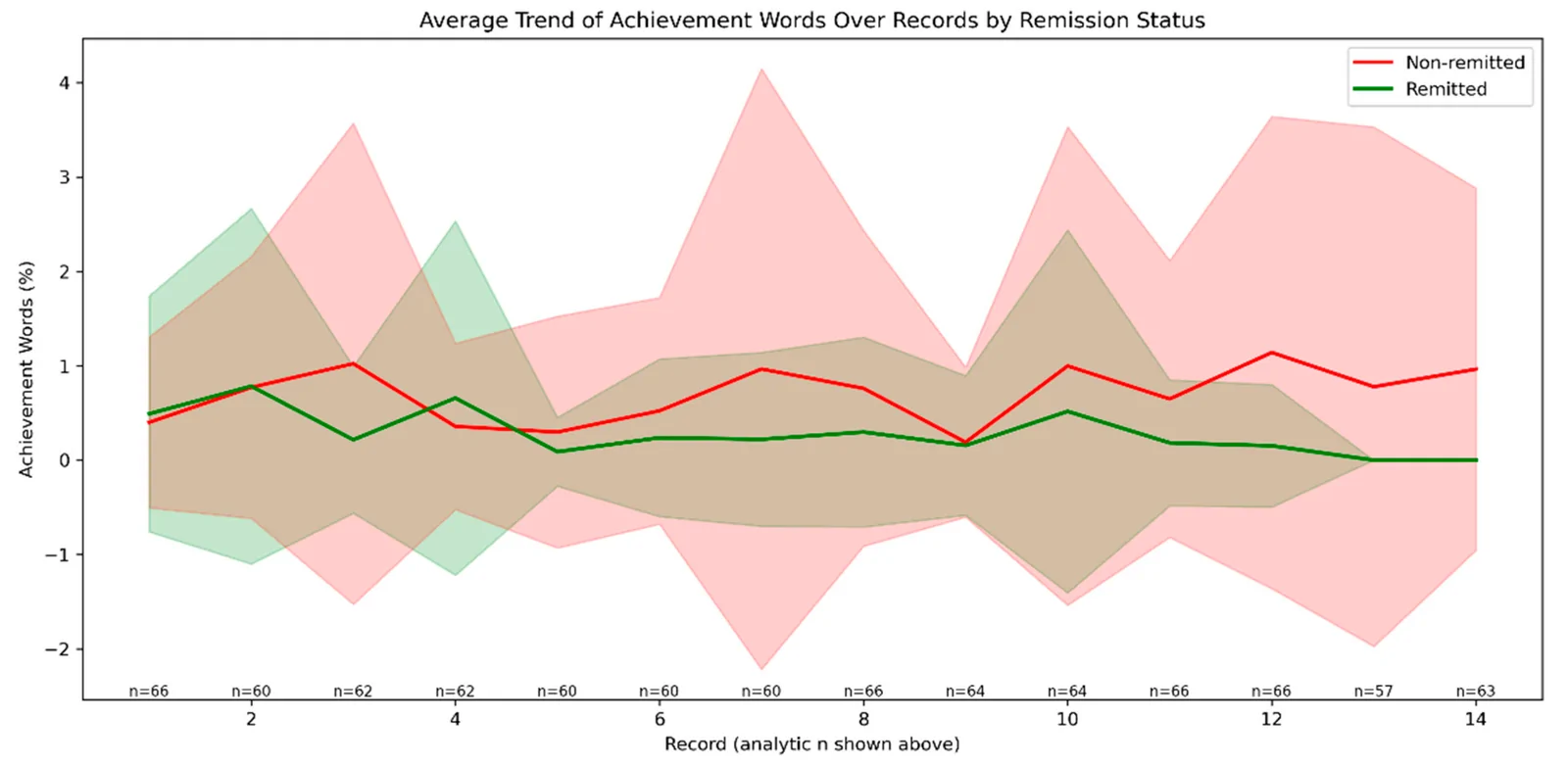
Trend analysis of achievement-related words between remitted and non-remitted individuals (the solid lines represent the mean feature values at each recording time point, and the shaded areas indicate ±1 standard deviation).

**Figure 4:**
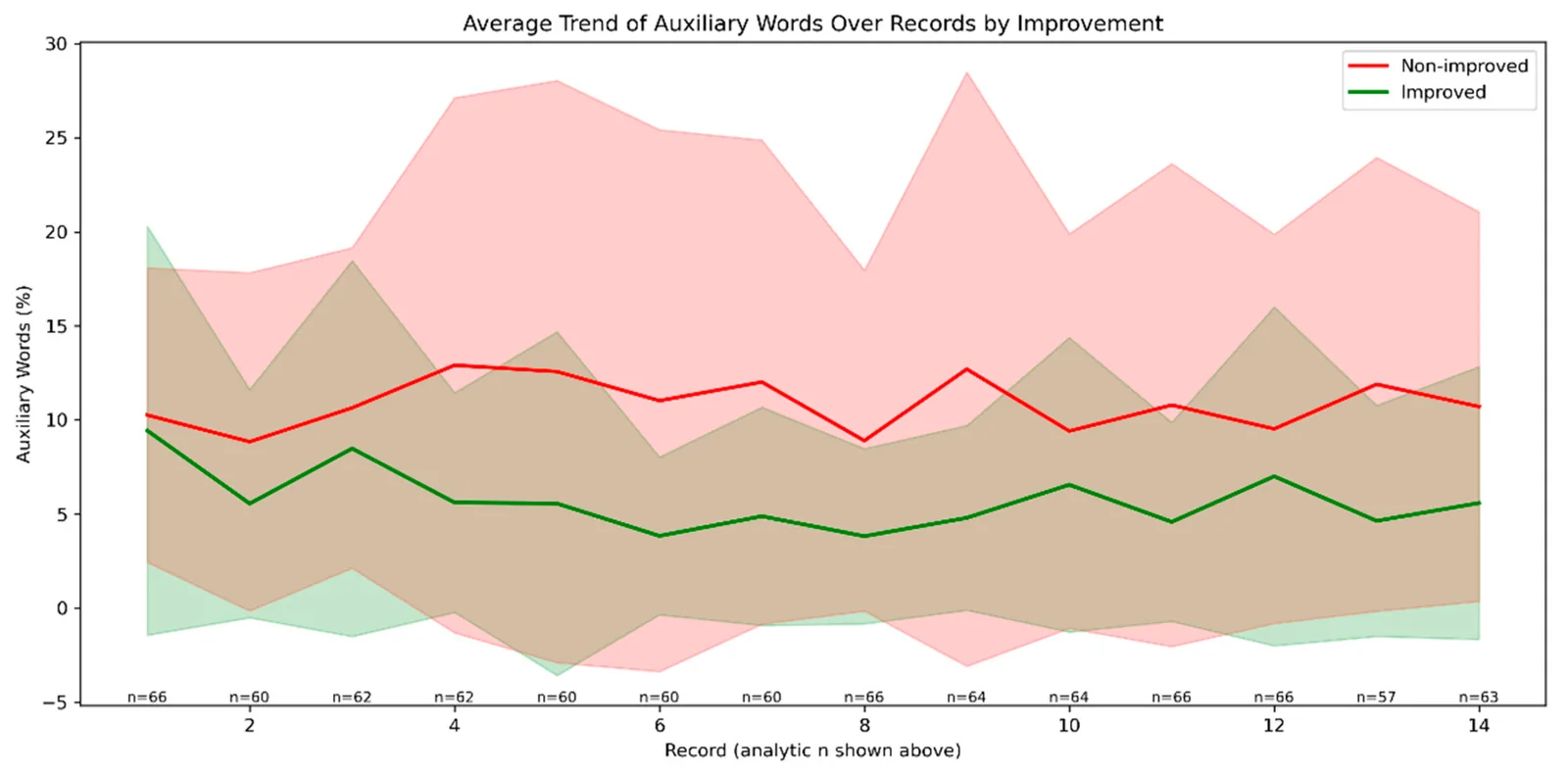
Trend analysis of auxiliary words between improved and non-improved individuals (the solid lines represent the mean feature values at each recording time point, and the shaded areas indicate ±1 standard deviation).

Longitudinal patterns of the significant linguistic features indicated overall decreases in achievement and auxiliary word use across the seven data collection time points. Achievement words showed a significant downward trend among remitted participants (β = −0.15, 95% CI: −0.28 to −0.03, *p* = 0.01; Figure 3). Auxiliary word use was also found to decrease significantly among participants who experienced symptom improvement (β = −0.64, 95% CI: −1.27 to −0.003, *p* = 0.049; Figure 4).

### 3.4. Machine Learning Model Performance

Across the 12 machine learning models trained and tested, three models achieved an AUC of over 0.80 in predicting depression remission, and four achieved that threshold in predicting depression improvement, among which the best-performing models are Stochastic Gradient Descent for predicting remission (AUC = 0.88) and Multilayer Perceptron for predicting improvement (AUC = 0.86) (Table 5 and Table 6). The best-performing multimodal models demonstrated superior predictive performance compared with the best-performing demographic-, linguistic-, and acoustic-based models (Tables S1 and S2).

## 4. Discussion

### 4.1. Changes in Depressive Symptoms After Voice Journaling

This study examined whether a seven-day digital voice journaling was associated with reductions in depressive symptoms. Consistent with our first hypothesis, HAMD-17 scores were significantly lower at the post-journaling assessment than at baseline with a moderate effect size and showed improvement and even remission of depressive symptoms in over half of the participants. While smaller than the large effect size found in gold standard treatment of depression—psychotherapy with or without antidepressants [36]—such precursory results suggest a plausible benefit of voice journaling for depression, which can be considered in the development of future self-help tools. Controlled trials are needed to confirm its effectiveness.

### 4.2. Linguistic and Acoustic Markers of Depressive Symptom Improvement and Remission

Supporting our second hypothesis, exploratory analyses revealed that speech characteristics are potential markers of changes in depressive symptoms, as distinct linguistic and acoustic features were found to be associated with depressive symptom improvement and remission.

Several acoustic features may reflect physiological changes associated with depression and recovery. For example, F2 frequency increased among participants who improved or remitted. Prior studies have reported lower F2 in individuals with depression, potentially reflecting altered vocal tract dynamics resulting from increased muscle tension in the jaw, facial, and laryngeal regions [37,38]. Consistent with these findings, the higher F2 observed among remitted participants may indicate normalization of articulatory movement. Higher F3 frequency was also associated with depression remission, although the underlying mechanism remains unclear. While F3 has been linked to aspiration-related speech characteristics [39,40,41], further research is needed to clarify its role in depressive symptomatology and recovery.

Numerous linguistic features that decreased among participants who improved or remitted may reflect reductions in cognitive and emotional features commonly linked to depression. These included words referring to the past [16,42], consistent with the tendency for depressive rumination to focus on negative past experiences [43]; home-related words, which may reflect reductions in home-related stressors associated with depression [44,45]; sadness-related words [14,42], aligning with the central role of low mood in depression; and work-related words, given the established relationship between chronic work stress and depressive symptoms [46]. Auxiliary words were also used less frequently among participants who improved. However, evidence regarding auxiliary word use in depression remains mixed, with studies reporting no differences [47], decreased use [15], and increased use [16] among individuals with depression. Further research is needed to clarify their relationship with depressive symptomatology.

A decline in achievement-related words was observed among participants who improved or remitted. This finding contrasts with previous studies reporting either a negative association [14,48] or no association [49] between achievement-related language and depression. One possible explanation is that individuals with depression may become preoccupied with perceived failures and unmet goals, leading to more frequent references to achievements they believe they should have attained. Such patterns are consistent with evidence linking depression to negative self-focused rumination and attentional biases [50,51]. This interpretation may be particularly relevant in East Asian contexts, where achievement is often highly valued and success is frequently defined by culturally salient benchmarks such as academic performance and career attainment [52,53,54,55,56,57]. Within these cultural settings, achievement-related concerns may represent an important source of psychological distress and a recurring theme in depressive cognition. The observed reduction in achievement-related language among participants who improved or remitted may therefore reflect a diminished focus on perceived inadequacies and achievement-related stressors.

Although our findings suggest that individuals who improved or remitted used fewer function words overall, previous studies have reported inconsistent results, with some showing lower function word use among individuals with depression than among healthy controls [15,58]. Function words have been proposed to serve important social and interpersonal functions, and reduced use may therefore reflect diminished social engagement associated with depression [58]. However, the interpretation of function word frequency is complicated by the heterogeneity of this category, which encompasses multiple subcategories, including pronouns, determiners, prepositions, auxiliary verbs, and conjunctions. In addition, the composition of function word categories varies across languages and dictionary versions. For example, English includes articles within the function word category [59], whereas Chinese does not contain an equivalent article class. As a result, total function word frequencies may not be directly comparable across languages. Future studies may benefit from examining specific function word subcategories rather than aggregate counts to better understand their relationship with depressive symptomatology.

### 4.3. Machine Learning Performance

In line with our third hypothesis, machine learning models integrating linguistic and acoustic features demonstrated strong predictive performance in identifying post-journaling clinical outcomes. Three multimodal models achieved AUC values greater than 0.80, with the best-performing models attaining AUCs of 0.88 for remission and 0.86 for symptom improvement. Notably, the best-performing multimodal models outperformed the strongest demographic-, linguistic-, and acoustic-based models, indicating that integrating multiple data modalities enhanced predictive accuracy. Although no directly comparable studies have examined the prediction of depression improvement or remission from digital voice journaling using linguistic and acoustic features, these findings compare favorably with prior work, including models predicting depression remission following online psychotherapy using demographic and clinical variables (AUC = 0.69) [46], AI-based depression detection using voice biomarkers (AUC = 0.78) [60], and machine learning models predicting bipolar depression severity from linguistic and acoustic features (AUC = 0.76) [27].

### 4.4. Potential Clinical Implications and Future Research

The observed reduction in depressive symptoms during the seven-day voice journaling period provides preliminary evidence of within-participant symptom change. However, as this study did not include a control group, the observed changes cannot be attributed to voice journaling itself. If its effectiveness is established in future controlled studies, the self-guided digital format of voice journaling may offer advantages in scalability and accessibility, particularly in settings where mental health services are limited. Prospective randomized controlled trials in clinical populations are therefore needed to determine its therapeutic effectiveness and potential clinical utility.

Several of our predictive models in this pilot study exceeded the commonly cited threshold of AUC ≥ 0.80 for potential clinical utility, providing preliminary evidence of possible clinical implications [61]. Such initial findings suggest that linguistic and acoustic markers derived from digital voice journaling may have potential for monitoring symptom trajectories and distinguishing individuals with different post-journaling symptom outcomes. Future research with further model calibrations, study replication or external validation, and randomized controlled trials with larger sample sizes, can pave the way for evaluating the clinical utility and impact of such predictive models in supporting personalized care by informing decisions about when additional clinical assessment or intervention may be warranted.

### 4.5. Strengths and Limitations

Some strengths of our study include the use of clinician-administered instead of self- reported depression measures that enhance the clinical validity of outcome assessment, the integration of both linguistic and acoustic features that can build a more comprehensive picture of speech characteristics, adherence to the TRIPOD+AI reporting framework to ensure transparency and reproducibility, and the collection of longitudinal instead of cross-sectional data, which can capture individual trajectories over the study period.

This study has several limitations. First, the relatively small sample size may limit the representativeness of the youth population, especially with a female-dominant sample, and reduce the generalizability of the machine learning models due to the risk of overfitting. As a pilot study, future research should include larger samples and external validation to assess the robustness and generalizability of the findings. Second, acoustic features may have been influenced by uncontrolled recording conditions, as participants self-recorded their entries rather than using a standardized environment. Such variability may partly explain why some acoustic findings were inconsistent with the previous literature. Future studies could mitigate this issue by providing more detailed recording instructions, including guidance on microphone distance, background noise, and the use of headphones or external microphones. Third, some participants provided relatively brief responses, which may have limited the richness of the linguistic and acoustic analyses. Future studies could encourage longer recordings by incorporating additional prompts or structured questions. Fourth, given that Logistic Regression analyses were used for a large number of individual speech characteristics, the exploratory nature of such analyses increases the probability of Type I errors. Future studies should use multiple-testing corrections to minimize false-positive outcomes. Fifth, although using a baseline questionnaire score to predict the follow-up outcome of the same measure is common practice, this approach carries known statistical risks, including autocorrelation and regression to the mean. Finally, the absence of a control group precludes causal inference regarding the observed changes in depressive symptoms, which could also reflect natural fluctuation, repeated assessment, expectancy, or concurrent care. These promising pilot findings warrant further evaluation in a randomized controlled trial.

## 5. Conclusions

Our study yielded three main preliminary findings. First, depressive symptoms decreased within participants over the seven-day digital voice journaling period, indicating short-term within-participant symptom change and supporting further investigation of digital voice journaling in controlled studies. Second, several linguistic and acoustic features were linked to depressive symptom improvement and remission. Differences from previous studies may reflect variations in recording conditions, language, and cultural context, emphasizing the need for context-sensitive interpretation of speech-based markers. Third, machine learning models showed strong initial performance in predicting depressive symptom improvement and remission, supporting their potential use for symptom monitoring and identifying individuals who may benefit from additional support. Further research with larger samples, external validation, and controlled designs is needed to confirm these findings and evaluate their clinical utility.

## Figures and Tables

**Table 1:** Summary of demographics and questionnaire scoring of eligible participants.

	Total (*n* = 68)		Improvement			Remission	
		Improved (*n* = 42)	Non-Improved (*n* = 26)	*p*	Remitted (*n* = 36)	Non-Remitted (*n* = 32)	*p*
Age (year), mean ± SD	20.78 ± 1.98	20.73 ± 2.08	20.85 ± 1.85	0.81	20.69 ± 2.07	20.88 ± 1.91	0.70
Female, n (%)	58 (85.29%)	35 (83.33%)	23 (88.46%)	0.73	30 (83.33%)	28 (87.50%)	0.74
Education, n (%)				0.29			0.29
- Secondary school	9 (13.24%)	4 (9.52%)	5 (19.23%)		3 (8.33%)	6 (18.75%)	
- Tertiary degree	59 (86.76%)	38 (90.48%)	21 (80.77%)		33 (91.67%)	26 (81.25%)	
SCID-5, n (%)							
- Any disorders	47 (69.12%)	27 (64.29%)	20 (76.92%)	0.41	21 (58.33%)	26 (81.25%)	0.08
- Depressive disorders	39 (57.35%)	23 (54.76%)	16 (61.54%)	0.77	17 (47.22%)	22 (68.75%)	0.12
- Anxiety disorders	30 (44.12%)	15 (35.71%)	15 (57.69%)	0.13	10 (27.78%)	20 (62.50%)	0.008 **
HAMD-17							
- Pre-journaling	13.00 ± 5.12	12.74 ± 5.12	13.42 ± 5.19	0.27	11.08 ± 3.00	15.16 ± 6.12	0.002 **
- Post-journaling	8.46 ± 6.27	5.21 ± 4.10	13.69 ± 5.61	<0.001 ***	3.81 ± 2.03	13.69 ± 5.18	<0.001 ***

SCID-5 = Structured Clinical Interview for DSM-5 Disorders–clinician version; HAMD = Hamilton Depression Rating Scale; ** *p* < 0.01; *** *p* < 0.001.

**Table 2:** Changes in HAMD-17 depression severity categories from pre- to post-journaling.

			Post-Journaling		
Pre-Journaling	Remission (HAMD-17 ≤ 7)	Mild (HAMD-17 = 8–16)	Moderate (HAMD-17 = 17–23)	Severe (HAMD-17 ≥ 24)	Total
Mild	34	19	1	1	55
Moderate	2	3	4	0	9
Severe	0	2	1	1	4
Total	36	24	6	2	68

**Table 3:** Linguistic and acoustic variables that are statistically significant in predicting depression remission in exploratory binary regression analyses, adjusted for age, gender and baseline HAMD-17 score.

Type	Predictor	Odds Ratio [95% CI]	*p*
Linguistic	Achievement words	0.19 [0.04, 0.61]	0.02 *
	Function words	0.94 [0.88, 0.99]	0.04 *
	Impersonal pronouns	0.37 [0.14, 0.88]	0.03 *
	Past-tense markers	0.06 [0.01, 0.65]	0.03 *
	Home-related words	0.62 [0.38, 0.94]	0.03 *
	Work-related words	0.66 [0.43, 0.97]	0.04 *
Acoustic	F2 frequency	1.02 [1.00, 1.03]	0.02 *
	F3 frequency	1.01 [1.00, 1.03]	0.02 *
	Loudness	0.06 [0.00, 0.71]	0.04 *
	MFCC 2	1.20 [1.04, 1.43]	0.02 *

MFCC = Mel-Frequency Cepstral Coefficients; * *p* < 0.05.

**Table 4:** Linguistic and acoustic variables that are statistically significant in predicting depression improvement in exploratory binary regression analyses, adjusted for age, gender and baseline HAMD-17 score.

Type	Predictor	Odds Ratio [95% CI]	*p*
Linguistic	Achievement words	0.34 [0.11, 0.84]	0.03 *
	Auxiliary words	0.92 [0.85, 0.99]	0.048 *
	Function words	0.94 [0.88, 0.99]	0.03 *
	Sadness words	0.44 [0.21, 0.82]	0.02 *
Acoustic	F2 frequency	1.01 [1.00, 1.03]	0.03 *
	MFCC 2	1.18 [1.03, 1.39]	0.03 *

MFCC = Mel-Frequency Cepstral Coefficients; * *p* < 0.05.

**Table 5:** Performance of machine learning models in predicting depression remission.

Machine Learning Model	AUC [95% CI]	*p*	Accuracy	Sensitivity	Specificity	PPV	NPV
Decision Tree	0.68 [0.56, 0.80]	0.002 **	0.66	0.61	0.72	0.71	0.62
Random Forest	0.75 [0.63, 0.87]	<0.001 ***	0.69	0.75	0.63	0.69	0.69
Support Vector Machines	0.40 [0.27, 0.52]	0.95	0.50	0.39	0.63	0.54	0.48
K-Nearest Neighbors	0.75 [0.63, 0.86]	<0.001 ***	0.66	0.81	0.50	0.64	0.70
Logistic Regression	0.87 [0.77, 0.94]	<0.001 ***	0.81	0.83	0.78	0.81	0.81
Stochastic Gradient Descent	0.88 [0.79, 0.95]	<0.001 ***	0.82	0.86	0.78	0.82	0.83
Multilayer Perceptron	0.87 [0.77, 0.95]	<0.001 ***	0.79	0.83	0.75	0.79	0.80
Gaussian Process	0.59 [0.47, 0.71]	0.08	0.65	0.75	0.53	0.64	0.65
AdaBoost	0.78 [0.68, 0.89]	<0.001 ***	0.68	0.75	0.59	0.68	0.68
Gradient Boosting	0.79 [0.66, 0.89]	<0.001 ***	0.69	0.83	0.53	0.67	0.74
Naive Bayes	0.75 [0.64, 0.85]	<0.001 ***	0.68	0.94	0.38	0.63	0.86
Quadratic Discriminant Analysis	0.66 [0.52, 0.79]	0.02 *	0.57	0.39	0.78	0.67	0.53

AUC: area under curve; * *p* < 0.05; ** *p* < 0.01; *** *p* < 0.001.

**Table 6:** Performance of machine learning models in predicting depression improvement.

Machine Learning Model	AUC [95% CI]	*p*	Accuracy	Sensitivity	Specificity	PPV	NPV
Decision Tree	0.51 [0.37, 0.65]	0.48	0.49	0.50	0.46	0.60	0.36
Random Forest	0.65 [0.51, 0.78]	0.02 *	0.66	0.88	0.31	0.67	0.62
Support Vector Machines	0.78 [0.66, 0.88]	<0.001 ***	0.71	0.83	0.50	0.73	0.65
K-Nearest Neighbors	0.75 [0.62, 0.86]	<0.001 ***	0.75	0.98	0.39	0.72	0.91
Logistic Regression	0.84 [0.73, 0.92]	<0.001 ***	0.69	0.81	0.50	0.72	0.62
Stochastic Gradient Descent	0.84 [0.74, 0.93]	<0.001 ***	0.81	0.82	0.73	0.84	0.76
Multilayer Perceptron	0.86 [0.76, 0.94]	<0.001 ***	0.77	0.76	0.77	0.84	0.67
Gaussian Process	0.53 [0.42, 0.66]	0.30	0.63	0.81	0.35	0.67	0.53
AdaBoost	0.66 [0.51, 0.80]	0.02 *	0.65	0.76	0.46	0.70	0.55
Gradient Boosting	0.74 [0.60, 0.86]	0.002 **	0.71	0.88	0.42	0.71	0.69
Naive Bayes	0.85 [0.75, 0.94]	<0.001 ***	0.79	0.83	0.73	0.83	0.73
Quadratic Discriminant Analysis	0.54 [0.43, 0.66]	0.25	0.59	0.74	0.35	0.65	0.45

AUC: area under curve; * *p* < 0.05; ** *p* < 0.01; *** *p* < 0.001.

## Data Availability

The pre-processed data, analysis code and package environment presented in this study are available on request from the corresponding author; however, raw recordings are unavailable due to ethical and privacy restrictions related to sensitive voice recordings and mental health data.

## Supplementary Materials

The following supporting information can be downloaded at https://www.mdpi.com/article/10.3390/healthcare14193238/s1, Supplementary Data S1: Completed TRIPOD+AI checklist. Supplementary Data S2: Completed TRIPOD+AI Abstract Checklist. Table S1: Performance of machine learning models in predicting depression remission. Table S2: Performance of machine learning models in predicting depression improvement.

## Abbreviations

The following abbreviations are used in this manuscript:

NLP	Natural Language Processing
HAMD-17	17-item Hamilton Depression Rating Scale
SCID-5-CV	Structured Clinical Interview for DSM-5 Disorders–Clinician Version
TRIPOD+AI	Transparent Reporting of a Multivariable Prediction Model for Individual Prognosis or Diagnosis using Artificial Intelligence
MFCC	Mel-Frequency Cepstral Coefficients
CLIWC	Chinese Linguistic Inquiry and Word Count Dictionary 2015
SD	Standard Deviation
RMSSD	Root Mean Square of Successive Differences
SMOTE	Synthetic Minority Over-Sampling Technique
AUC	Area Under the Receiver Operating Characteristic Curve
PPV	Positive Predictive Value
NPV	Negative Predictive Value
OR	Odds Ratio
CI	Confidence Interval
GEE	Generalized Estimating Equation
